# Cutaneous leiomyosarcoma: a 20-year retrospective study and review of the literature^☆^^☆☆^

**DOI:** 10.1016/j.abd.2020.10.003

**Published:** 2021-03-21

**Authors:** Catarina Soares Queirós, Paulo Filipe, Luís Soares de Almeida

**Affiliations:** a Dermatology Department, Hospital de Santa Maria, Centro Hospitalar e Universitário de Lisboa Norte, Lisbon, Portugal; b Faculty of Medicine, Universidade de Lisboa, Lisbon, Portugal

**Keywords:** Immunohistochemistry, Leiomyosarcoma, Pathology, Skin neoplasms

## Abstract

**Background:**

Cutaneous leiomyosarcoma is a rare malignant neoplasm with muscular origin, representing 2%-3% of all cutaneous soft tissue sarcomas.

**Objectives:**

The aim of this study was to characterize clinicopathological features of patients diagnosed with cutaneous leiomyosarcoma in our center over the last 20-years.

**Methods:**

A retrospective study of patients with a histopathological diagnosis of leiomyosarcoma between 1999 and 2018 was conducted.

**Results:**

Eleven patients were diagnosed with cutaneous leiomyosarcoma during this period. Most cases occurred in men (n = 7). Age at presentation ranged from 47 to 92 years (mean 64.9 years). Head and neck were the most frequently involved locations (n = 5). Ten leiomyosarcoma were dermal, with one cutaneous metastasis. Immunohistochemical staining was available for 7 patients, demonstrating positivity for smooth muscle actin in all of them. All neoplasms were treated surgically. Mean survival was 32.2-months.

**Study limitations:**

This was a retrospective study based on medical and pathological records.

**Conclusions:**

Histopathology is essential for the diagnosis of leiomyosarcoma, usually revealing a dermal or subcutaneous lesion composed of intertwined fascicles of smooth muscle fibers. Immunohistochemistry is then used to adequately differentiate leiomyosarcoma from other spindle cell tumors. When dealing with cutaneous leiomyosarcoma, it is advisable to carefully evaluate the depth of subcutaneous extension, since even minimal subcutaneous involvement may be associated with a poorer prognosis.

## Introduction

Cutaneous Leiomyosarcoma (LMS) is a rare malignant neoplasm with a muscular origin, representing around 2%–3% of all cutaneous soft tissue sarcomas and 0.04% of all neoplasms.^1^ On the skin, leiomyosarcoma is the third in frequency, behind dermatofibrosarcoma protuberans and Kaposi's sarcoma.^2^

Traditionally, cutaneous LMS has been classified into three clinicopathological groups, with different prognostic implications. These include purely skin or dermal forms, subcutaneous or hypodermic forms, and cutaneous metastases of extracutaneous leiomyosarcoma.^1^ As a rule, the prognosis worsens as the lesions deepen.^2^

Dermal leiomyosarcomas seem to arise from dermal piloerector smooth muscle.^3^ This type of leiomyosarcomas generally behaves as a non-aggressive neoplasm, with a relative tendency to local recurrence after removal (24%) but with a low risk of distant metastasis (3%–14%).^1^, ^4^, ^5^ On the other hand, subcutaneous leiomyosarcomas derive from smooth muscle fibers of the middle layer of the vascular wall of arteries and veins.^3^ Unlike the superficial or dermal variant, they are characterized by a high rate of locoregional recurrence (37%) and distant metastasis (21%–62%), thus considered to have a less favorable prognosis.^5^ Lastly, cutaneous leiomyosarcoma metastases represent an unfortunate indicator of the progression from the primary tumor, usually originated in the retroperitoneum, the uterus or the subfascial plane of the extremities, although they can arise from primary dermal or subcutaneous forms.^1^, ^2^ The prognosis of these patients is usually poor, with a mean survival of 16-months after metastasis appearance.^5^

Histologically, LMS is characterized by a dermal proliferation of elongated spindle-shaped cells arranged in interweaving fascicles with blunt-ended, cigar-shaped nuclei and eosinophilic cytoplasm. Mitotic figures are usually easily identifiable.^1^

Most of the literature published to date on cutaneous LMS refers to case reports or small case series, thus highlighting the difficulty in characterizing its heterogeneous clinical behavior and prognostic factors and leaving many questions unanswered. The aim of this study was to characterize clinicopathological features of patients diagnosed with cutaneous LMS in our center over the last 20-years.

## Methods

A retrospective study of patients with a histopathological diagnosis of cutaneous LMS was conducted. Patients between the 1^st^ January 1999 and the 31^st^ December 2018 were included. Cases were collected from the database of the Department of Cutaneous Pathology of the Santa Maria Hospital and the Lisboa Norte Hospital and University Center, Portugal.

In this group of patients, the following clinical variables were collected: age of presentation, sex, year of diagnosis, clinical presentation (the type of lesions, number, size, and location), time from diagnosis of the primary tumor until the detection of cutaneous metastasis, follow-up period, evolution, and response to the treatments. Regarding histological findings, the histological variant (dermal, hypodermic, or metastatic) and the positivity against several immunohistochemical stains (vimentin, desmin, muscle-specific actin (HHF-35), smooth muscle actin (SMA), H-caldesmon, S100, and CD34) were collected.

## Results

Overall, eleven patients were diagnosed with cutaneous LMS during the considered 20-year period. Most cases occurred in men (n = 7), with 4 cases in women. Age at presentation ranged between 47- and 92-years (mean age 64.9-years; standard deviation 14.6-years). Head and neck were the most frequently involved locations (n = 5), followed by the lower extremity (n = 4) and trunk (n = 2).

Seven patients complained of a painful pinkish nodule, the rest of them showing asymptomatic lesions. The majority of the tumors were dermal LMS (n = 10), with only one extracutaneous LMS (metastasis of an LMS of the deep femoral vein).

Immunohistochemical staining was available for 7-patients, demonstrating positivity for smooth muscle actin in all of them and either positivity for desmin or vimentin. H-caldesmon was positive in 3 cases and HHF-35 in one case.

All neoplasms were treated surgically, with 2 to 5 cm margins. Only one patient required adjuvant radiotherapy. Mean survival was 32.2-months (ranging from 3- to 108-months).

## Discussion

Cutaneous soft tissue sarcomas, which include LMS, represent a group of rare neoplasms in clinical practice. The incidence rates of LMS, in particular, have been estimated at 0.2/100,000/year, entailing an added difficulty in their diagnosis and treatment.^1^, ^3^

Dermal leiomyosarcoma is thought to originate from arrector pili (smooth) muscle cells. Notably, anecdotal cases originated in the smooth muscle of the genital dartos or its vulvar equivalent and the mammary areola have been reported. Subcutaneous LMS seems to be derived from vascular (smooth) muscle cells. On the molecular level, receptor tyrosine kinase (such as IGFR and PDGFR) overexpression has been demonstrated.^3^

Cutaneous LMS usually manifests as nodules or masses with a firm consistency, in whites over 50-years of age.^2^ Most cutaneous LMS occur in the fifth to seventh decades of life, with subcutaneous LMS usually occurring in patients aged 50- to 80-years.^6^ Because the subcutaneous LMS tend to be more aggressive tumors and are usually diagnosed at a more advanced stage, they are usually larger than cutaneous LMS. Men seem to be affected more often than women (ratio 3:1), a finding that is concordant with our series.^2^, ^6^

The etiology of these tumors remains unknown, although predisposing factors for cutaneous LMS include leiomyomas as precancerous lesions and history of previous injury or local trauma. However, most cutaneous LMS originate again, with no prior triggering factor.^2^ Indeed, none of our patients experienced physical trauma, precancerous lesions, or previous radiation exposure.

Approximately 50% of cutaneous LMS are located on the extensor surfaces of the lower extremities, showing a predilection to areas with a greater density of hair follicles and arrector pili muscles, such as the extensor surfaces.^2^ Less frequently, lesions have been described on the scalp and face and even trunk, lip, genital (scrotum, vulva, penis), and gluteal area.^2^ In our material, lesions favored the head and neck region, followed immediately by the lower extremities.

The clinical presentation of cutaneous LMS is non-specific, the most frequent being a single firm pink nodule with a smooth surface or a more exophytic tumor with reddish or brown coloration. Subcutaneous lesions seem better delimited than dermal ones and remind a lipoma, but with more solid consistency. Plate-type LMS has been described, characterized by multiple grouped indurated nodules.^2^ It is important to highlight that before making a definitive diagnosis of a primary cutaneous LMS, one has to rule out the possibility of a metastatic cutaneous LMS from an LMS of deep or visceral tissues, particularly when facing of subcutaneous or multiple nodules.^2^ Given their clinical manifestation, epidermal cysts and cutaneous metastases are important differential diagnoses.^3^

Dermal LMS is usually in the form of slow-growing lesions, with size at diagnosis between 1 and 3.5 cm; subcutaneous LMS are usually larger. Pain during palpation is frequent (63% of cases), as we could confirm in our series, and sometimes can occur even without handling. Other associated symptoms include itching, burning and paresthesias in the territory of the lesion.^1^, ^2^

**Figure 1:**
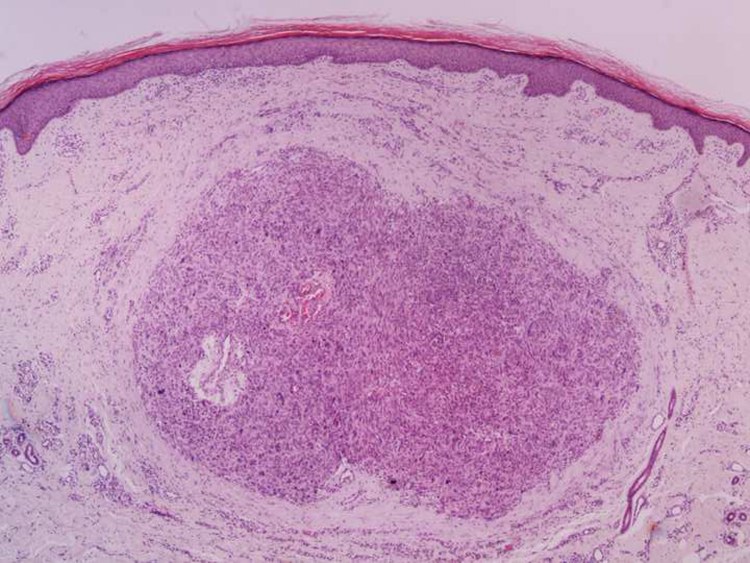
Dermal leiomyosarcoma – low power field. Nodular lesion located in the dermis, non-encapsulated, and without connection with the adjacent epidermis (Hemathoxylin & eosin, ×40).

**Figure 2:**
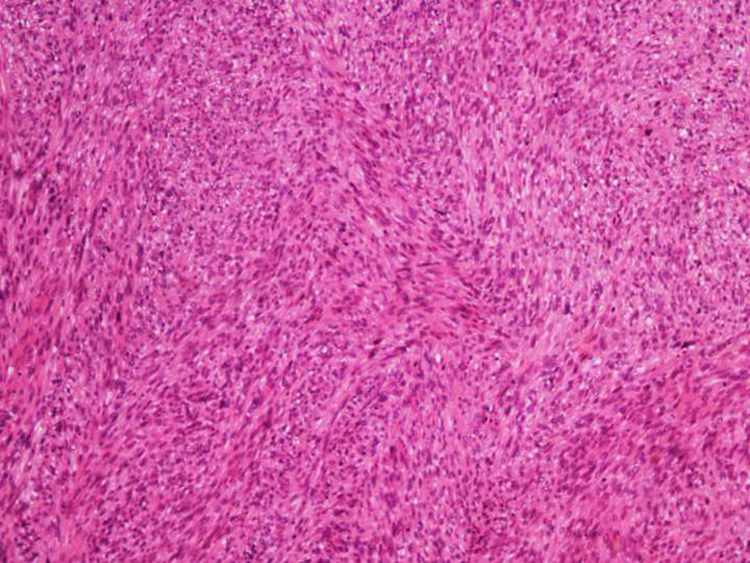
Dermal leiomyosarcoma – medium power field. The tumor is composed of intertwined fascicles of smooth muscle fibers (Hemathoxylin & eosin, ×100).

**Figure 3:**
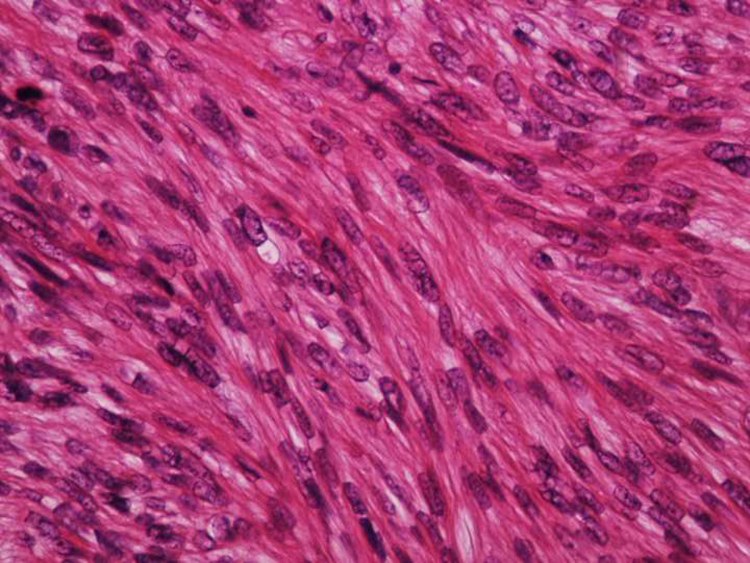
Dermal leiomyosarcoma – high power field. The cells are spindle-shaped, with elongated nuclei and blunt ends, inconspicuous nucleolus, and fibrillary eosinophilic cytoplasm (Hemathoxylin & eosin, ×400).

It is essential for the diagnosis to perform a biopsy that includes subcutaneous cellular tissue. In the histological study, dermal LMS is usually a poorly defined lesion that occupies the entire thickness of the dermis and sometimes infiltrates the cellular subcutaneous tissue. Subcutaneous LMS is a better delimited and circumscribed lesion that compresses adjacent tissue and is located entirely in the hypodermis, with preservation of the dermis. In both cases, the lesions seem to be composed of intertwined fascicles of smooth muscle fibers. The cells are spindle-shaped, with elongated nuclei and blunt ends, inconspicuous nucleolus, and fibrillar eosinophilic cytoplasm. Some cells have a clear perinuclear halo characteristic of the muscle cell (Figure 1, Figure 2, Figure 3).^2^ The degree of nuclear pleomorphism is variable, mitosis is usually present (>1 mitosis per high-power field) and necrosis can be present or absent.^4^

From a histopathological point of view, two architectural patterns have been described: the nodular pattern, characterized by greater cellularity, atypia and mitotic figures, and the diffuse pattern, which is less cellular and with a lower mitotic burden. Occasionally, there is no obvious cellular atypia, making a differential diagnosis with leiomyoma difficult. In these instances, the histological diagnosis should be based on the overall architecture of the lesion, with an infiltrative pattern and increased cellularity being suggestive of malignancy. Rare histopathological variants of LMS include LMS of epithelioid cells, LMS of multinucleated giant cells, LMS of granular cells, sclerotic LMS, and pleomorphic and myxoid variants.^2^

**Figure 4:**
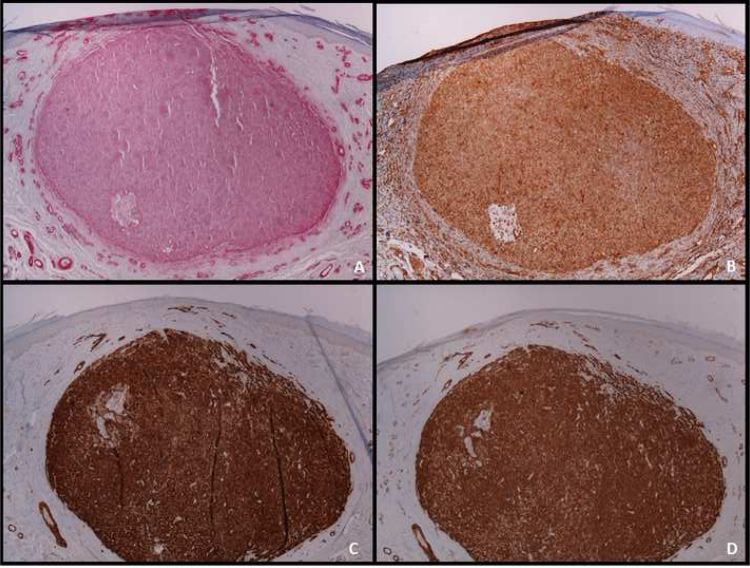
Immunohistochemistry of dermal leiomyosarcoma. A, Positive staining for smooth muscle actin (×40); B, Positive staining for vimentin (×40); C, Positive staining for H-caldesmon (×40); D, Positive staining for HHF-35 (×40).

An immunohistochemical study is a fundamental tool for the adequate differential diagnosis between LMS and other spindle cell tumors with similar histological characteristics. The well-differentiated LMS is positive to vimentin, desmin, h-caldesmon, muscle-specific actin, alpha-smooth muscle actin, and smooth muscle myosin (Fig. 4). In more undifferentiated lesions and subcutaneous LMS, desmin is frequently negative. To establish the diagnosis, at least two immunohistochemical muscle markers are necessary. Sometimes S-100 protein is positive, as well as cytokeratins. Other immunohistochemical stains performed to rule out other spindle-cell lesions (spindle cell carcinoma, desmoplastic melanoma, dermatofibrosarcoma protuberans, nodular fasciitis, malignant peripheral nerve sheath tumor, spindle cell atypical fibroxanthoma, fibrosarcoma, synovial sarcoma, and vascular tumors) include EMA, CD34, CD117, CEA, HMB45, Mart-1, Melan A and CK7; all of them are negative in LMS.^2^ Diffuse overexpression of p53 has been described to occur in cutaneous LMS but not in leiomyomas, being helpful in the differential diagnosis between these two conditions.^6^

The tumor-node-metastasis staging system of the American Joint Committee on Cancer (AJCC) is the standard sarcoma staging system and is also applied to cutaneous LMS. The prognosis of LMS depends on whether it is a cutaneous or subcutaneous lesion. As previously stated, dermal LMS has a tendency to local recurrence but a low probability of metastasis; on the other hand, subcutaneous LMS is more prone to local recurrence and metastases. Therefore, stratification and follow-up are different for these two forms.^2^

The utility of preoperative imaging for cutaneous LMS is not established, but several recommendations and diagnostic algorithms have been proposed, depending on the clinical and histopathological characteristics of the tumor.^1^ Patients with a dermal LMS should perform a magnetic resonance of the affected area before surgery, especially in the case of large or infiltrated lesions or those in locations difficult to access, such as the head. Although there are no studies, it is possible that ultrasound can replace magnetic resonance in this evaluation. In addition, a chest x-ray prior to surgery should be included. In subcutaneous LMS it is always advisable to perform a magnetic resonance of the area before surgery and a thoracoabdominal computed tomography to rule out the possibility of a metastatic cutaneous LMS from a deep tissue LMS.^1^, ^2^

Complete surgical excision is the treatment of choice for cutaneous LMS. However, one of the most important limitations in the approach to these neoplasms is the difficulty in defining the appropriate safety margins, where no consensus currently exists. Classic recommendations suggested aggressive surgeries with margins of 3–5 cm; however, similar results have recently been observed with more conservative surgeries (margins of 1 cm), without higher local recurrence rates and with improved morbidity.^7^ The deep margin should reach up to the fascia, and in more infiltrating cases the muscle should be included.^7^ Evaluation of surgical margins is a key-point in establishing the probability of future recurrence. Therefore, all pathology reports should include such data, as well as the shortest distance to the nearest surgical margin.^4^ If surgical margins are not clearly negative, re-excision should be undertaken if possible. It is estimated that up to 20% of patients may require repeated excisions to achieve negative margins.^7^ Mohs micrographic surgery may be of particular interest in cutaneous LMS.^6^

The role of adjuvant radiotherapy is not clearly defined, but this may have relevance in deep or large lesions (greater than 5 cm), in cases associated with poor prognostic factors, or in the setting of positive surgical margins, if a new resection is not possible.^7^, ^8^ In unresectable cases, radiation with or without chemotherapy is recommended, although success rates are mixed. Radiotherapy can also be used in local palliative control in cases with metastasis.^7^

Distant metastases are most commonly observed in the lungs, but superficial LMS may also metastasize to other cutaneous sites, namely to the scalp; scalp metastases may even be a harbinger of poor outcomes.^3^, ^6^ When facing of disseminated disease, there is currently indication for neoadjuvant, adjuvant, or palliative chemotherapy, usually based on traditional chemotherapeutic regimens used for connective and soft tissue sarcomas.^1^ The most commonly used agents include doxorubicin and ifosfamide, gemcitabine, docetaxel, taxotere, dacarbazine, and trabectedin; although not curative, these treatments have shown to delay disease progression.^3^, ^9^

Given the low incidence of cutaneous LMS, it is recommended that the treatment is carried out in a center specialized in sarcomas. Advanced therapies, such as immunotherapy and gene therapy, are being developed. Targeted therapies using tyrosine kinase inhibitors are also currently being investigated in clinical trials.^3^

There are no standard guidelines for LMS follow-up, but a clinical examination is recommended every 4 months during the first two years, for the early detection of possible local recurrences. Thereafter, controls every 6 months until the fifth year after surgery is advised; subsequently, once a year until 20 years, since very late relapses have been described.^4^, ^6^ There are also no recommendations for radiological examinations in the postoperative follow-up of patients with cutaneous sarcomas. However, the practice of a simple annual chest radiograph in the first five years after surgery and clinical evaluation of the surgical bed and locoregional lymph nodes seems like a reasonable strategy.^9^ In some cases, magnetic resonance may be helpful, especially in recurrent or hypodermic lesions or in those cases where surgery has been complex. Metastases are produced mainly by a hematogenous route to the lung, skin, and, less frequently, to regional lymph nodes. The appropriate staging test for patients suspected of disseminated disease is thoracoabdominal computed tomography.^9^

Several studies have evaluated prognostic factors in cutaneous LMS. In a multivariate analysis from Jensen et. al, only tumor size was shown to be an independent prognostic factor in relation to decreased survival.^10^ Factors such as a tumor size equal to or greater than 5 cm, deep location with fascia involvement, and a high histological grade were correlated with decreased survival in a univariate analysis.^10^ In 2007, Svarvar et. Al. observed that tumor depth was a significant prognostic factor for the development of metastasis, being also a significant prognostic factor for local recurrence and death.^8^ The strongest risk factor related to death in this study was the presence of metastases, either at presentation or subsequently. Moreover, these authors also showed that prognostic factors for the development of metastasis (and, consequently, for tumor-related death) differ from prognostic factors for local recurrence.^8^ In sum, location, size, and histologic grade (differentiation) seem to be the most important prognostic factors for cutaneous LMS.^4^, ^6^ As previously stated, the prognosis of dermal LMS is usually better, with 5-year-survival rates over 95%.^7^ Subcutaneous LMS has a greater tendency for recurrence and metastasis, therefore resulting in lower 5-year-survival rates of around 65%.^4^, ^8^

## Conclusion

In conclusion, cutaneous leiomyosarcoma is a low-grade malignancy with varying prognosis, depending on the location, size, and histological grade of the tumor. Histopathological examination is of the foremost importance in its diagnosis, usually supported by immunohistochemical studies. Favorable outcomes are possible to achieve after surgical resection with 1 cm surgical margins. In the face of recurrences or metastatic disease, radiotherapy and chemotherapy regimens may be employed, usually after a multidisciplinary discussion. When dealing with cutaneous leiomyosarcomas, it is advisable to carefully evaluate the depth of subcutaneous extension, since even minimal subcutaneous involvement may be associated with a poorer prognosis, thereby requiring a careful and prolonged clinical follow-up.

Although the small number of cases included and the retrospective nature of our study does not allow us to generalize the results obtained, our findings are concordant with the available literature and highlight the importance of early recognition and adequate excision of these tumors in order to improve prognosis.

## Financial support

None declared.

## Authors’ contributions

Catarina Soares Queirós: Data collection; data analysis; manuscript writing.

Paulo Filipe: Manuscript revision.

Luís Soares de Almeida: Data analysis; manuscript revision.

## Conflicts of interest

None declared.
